# Preventing Gestational Diabetes with a *Healthy Gut Diet*: Protocol for a Pilot, Feasibility Randomized Controlled Trial

**DOI:** 10.3390/nu15214653

**Published:** 2023-11-02

**Authors:** Nina Meloncelli, Hannah O’Connor, Shelley A. Wilkinson, Marloes Dekker Nitert, Lauren Kearney, Susan de Jersey

**Affiliations:** 1 Centre for Health Services Research, Faculty of Medicine, The University of Queensland, Brisbane, QLD 4029, Australia; 2 Office of the Chief Allied Health Practitioner, Metro North Hospital and Health Service, Brisbane, QLD 4029, Australia; 3 Department of Obstetric Medicine, Mater Mothers Hospital, South Brisbane, QLD 4101, Australia; shelley.wilkinson@mater.org.au; 4 Faculty of Health and Behavioural Sciences, School of Pharmacy, The University of Queensland, Brisbane, QLD 4102, Australia; 5 School of Chemistry and Molecular Biosciences, The University of Queensland, St. Lucia, QLD 4072, Australia; m.dekker@uq.edu.au; 6 Women’s and Newborn Service Group, Royal Brisbane and Women’s Hospital, Metro North Health, Brisbane, QLD 4029, Australia; lauren.kearney@uq.edu.au; 7 School of Nursing, Midwifery and Social Work, The University of Queensland, Brisbane, QLD 4102, Australia; 8 Department of Dietetics and Foodservices, Royal Brisbane and Women’s Hospital, Metro North Health, Brisbane, QLD 4029, Australia

**Keywords:** gestational diabetes mellitus, gut microbiota, dietary intervention, pregnancy, study protocol, behavior change techniques

## Abstract

Around 14% of pregnancies globally are affected by gestational diabetes mellitus (GDM), making it one of the most common disorders experienced by women in pregnancy. While dietary, physical activity and supplement interventions have been implemented to prevent GDM, with varying levels of success, altering the gut microbiota through diet is a promising strategy for prevention. Several studies have demonstrated that women with GDM likely have a different gut microbiota to pregnant women without GDM, demonstrating that the gut microbiota may play a part in glycemic control and the development of GDM. To date, there have been no randomized controlled trials using diet to alter the gut microbiota in pregnancy with the aim of preventing GDM. Here, we present the study protocol for a single-blind randomized controlled trial which aims to determine the effectiveness of the *Healthy Gut Diet* on reducing the diagnosis of GDM in pregnant women with one or more risk factors. Consenting women will be randomized into either the *Healthy Gut Diet* intervention group or the usual care (control) group after 11 weeks gestation. The women in the intervention group will receive three telehealth counseling appointments with an Accredited Practicing Dietitian with the aim of educating and empowering these women to build a healthy gut microbiota through their diet. The intervention was co-designed with women who have lived experience of GDM and incorporates published behavior change techniques. The control group will receive the usual care and will also be shown a brief (3 min) video on general healthy eating in pregnancy. The primary outcome is the diagnosis of GDM at any stage of the pregnancy. Secondary outcomes include changes to gut microbiota composition and diversity; gestational weight gain; maternal and infant outcomes; management of GDM (where relevant); dietary quality and intake; physical activity; and depression scoring. We aim to recruit 120 women over 16 months. Recruitment commenced in January 2023. The trial has been registered with the Australian New Zealand Clinical Trials Registry (ACTRN12622001285741).

## 1. Introduction

Gestational diabetes mellitus (GDM) is a common and costly condition that occurs during pregnancy. Globally, the prevalence of GDM is estimated to be around 14% [1]. In Australia, GDM incidence has doubled in the decade since 2010 and affects one in six Australian pregnancies [2,3]. Although much of this increase has been due to changes to the diagnostic criteria, certain risk factors increase the risk of developing GDM [4]. Up to 70% of women with GDM will develop it again in subsequent pregnancies [5]. The effects of GDM are intergenerational. Women with GDM have a 10-fold increased risk of type 2 diabetes [6] and a 2-fold risk of cardiovascular events [7]. Their children are more likely to develop cardiometabolic conditions and to experience obesity [8].

Medical nutrition therapy and physical activity, together with self-monitoring of blood glucose levels, are the cornerstone of GDM management; however, around 50% of women will require pharmacological intervention in the form of oral hypoglycemic agents or insulin [9]. Strong predictors of GDM include early excess gestational weight gain (GWG), a pre-pregnancy body mass index (BMI) above 25 kg/m^2^, and a history of GDM [5,10,11]. Several studies have demonstrated a positive effect of dietary intervention for the prevention of GDM [12] but have not consistently shown reductions in GDM risk [13].

The gut microbiota—the community of microorganisms in the human digestive system—play a role in glycemic control in GDM [14]. Although not consistently demonstrated in all studies, recent evidence shows that women with GDM may have significantly different microbiota compositions to women without GDM [15] with similarities to the non-pregnant population with type 2 diabetes [14,16,17,18,19]. The gut microbiota can be modified by a range of factors, including diet, dietary supplements, physical activity, pregnancy hormones, and antibiotics [20,21]. Although diet can rapidly alter the gut microbiota, particularly in the short term [21], few studies have investigated the link between diet, the maternal gut microbiota and the development of GDM. One observational study has shown that adherence to a standard GDM diet produced better metabolic and inflammatory outcomes, which are associated with significant differences in gut microbiota composition [22]. While it seems promising that dietary interventions designed to modulate the gut microbiota may produce favorable pregnancy outcomes, there is a clear lack of evidence on the impact of dietary interventions designed to improve gut microbiota composition and the development of GDM.

A healthy gut diet is abundant in fiber-rich, prebiotic whole foods found exclusively in vegetables, fruit, whole grains, legumes/lentils, nuts, and seeds [23]. A healthy maternal diet plays an influential role in epigenetic programming, affecting the health of the next generation [24]. Recent evidence shows that pregnant women counseled to ‘eat for their gut’ have improved diet quality [25]. Dietary interventions that target the maternal gut microbiota show potential to reduce the health burden associated with GDM. Although modulating the gut microbiota shows promise for the prevention of GDM [12], specific interactions between dietary components and changes to gut microbiota function in early pregnancy remain unexplored.

The primary aim of this study is to determine the effectiveness of a dietary intervention called ‘*The Healthy Gut Diet*’ on reducing the diagnosis of GDM in pregnant women with one or more specified risk factors. We hypothesize that there will be fewer women diagnosed with GDM in the intervention group and this effect will be enhanced amongst women who adhere closest to the dietary recommendations. The secondary aim is to understand whether dietary intervention can modulate the gut microbiota compared with the usual care group and if this effect is enhanced in women who closely adhere to the dietary intervention compared to those who are less adherent. The third aim is to compare the maternal and infant outcomes of women enrolled in the dietary intervention and those in the usual care group.

## 2. Materials and Methods

### 2.1. Design

**Figure 1:**
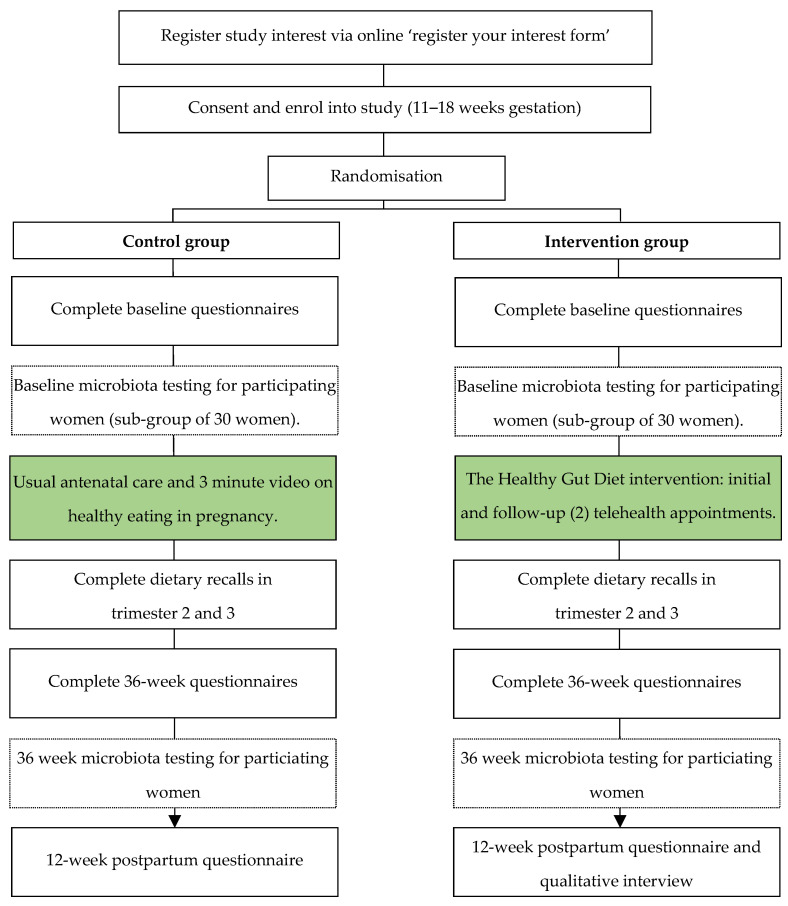
Flow chart for the control and intervention groups for the study on the Healthy Gut Diet with regards to preventing gestational diabetes.

This study is a pilot single-blind, two-arm, parallel randomized controlled trial conducted to test the effect of the dietary intervention for pregnant women at risk of developing GDM. We will aim to recruit 120 women in total, who will be randomly allocated into the intervention or control group. The study flow diagram is shown in Figure 1.

### 2.2. Setting

The study is open to all pregnant women who meet the criteria who are birthing at public or private maternity facilities in Queensland, Australia. Geographically, Queensland is the second-largest state/territory and third-most populous state in Australia. Between 60,000 and 62,000 babies are born in Queensland annually. Nationally, the incidence of GDM is 16% [2].

### 2.3. Eligibility Criteria

Pregnant women, 18 years or older, with a singleton pregnancy are eligible if they enroll in the study prior to 18 weeks gestation, and have one or more of the following risk factors for developing GDM: prior history of GDM; a first-degree relative with type 2 diabetes mellitus or a mother/sister with a history of GDM; pre-pregnancy body mass index (BMI) greater than 25 kg/m^2^; or a biological child who weighed more than 4000 g at birth.

### 2.4. Exclusion Criteria

Women will be excluded if they have a confirmed diagnosis of any form of diabetes (including GDM) at the time of enrolment. Due to the complex or confounding nature of certain conditions, women with reported cystic fibrosis, inflammatory bowel disease, bariatric surgery or bowel disorder requiring a restrictive diet, a diagnosed eating disorder, or other complex medical co-morbidities (for example, kidney or heart disease) will also be excluded. Assisted conception (for example in vitro fertilization) is not an exclusion criterion. Women who are unable to understand the intervention (e.g., have insufficient understanding of spoken English) are also excluded.

### 2.5. Recruitment

As this is a state-wide study, multiple recruitment strategies have been used. The primary strategies are ongoing, paid social media advertisements from the study’s Instagram and Facebook pages, targeting women between 18 and 50 years in Queensland. Antenatal care providers (obstetricians, general practitioners, and midwives) have also been targeted via study information, flyers and business-card sized ‘referral cards’ sent to public and private clinical and special interest networks and cold-contacting private practices via email and postal mail. This second strategy recognizes that one of the more successful recruitment strategies for trials is referrals from trusted health care professionals [26]. Women can also be identified upon referral to the study hospitals (two small metropolitan hospitals). Other recruitment strategies involve putting flyers in doctor’s surgeries, maternal and child health centers, childcare, playgroups, shopping centers, and toy libraries. Women who have consented to be contacted, either verbally at participating study sites or by completing the online ‘Register Your Interest’ form, are sent an SMS inviting them to participate in the study.

### 2.6. Randomisation and Blinding

Randomization of a 1:1 group allocation ratio with randomly permuted blocks was generated prior to the study recruitment commencing and stored in electronic sealed envelopes. Following an eligibility check, the study lead allocates each new enrolled participant by selecting the next ‘sealed envelope’. Participant blinding is not possible due to the nature of a dietary intervention. The researchers involved in data analysis will be blinded to the group allocation. Only the study lead, who will not be directly involved in data analysis, will be aware of the participant group allocation until analysis is completed.

### 2.7. Participation

Women in both groups receive an AUD 50 grocery store gift voucher after completing the baseline questionnaire and trimester 2 dietary intake records, and a second AUD 50 grocery store gift voucher will be received upon completion of the 36-week questionnaire and trimester 3 dietary intake records. All participants will be invited to complete a 12-week post-partum follow-up questionnaire and will receive a third AUD 25 grocery store gift voucher upon completion. Women in the intervention group will also be invited to participate in a short phone interview that will explore the barriers and enablers of the Healthy Gut Diet (both within pregnancy and longer term). Women who undertake gut microbiota analysis will receive a personalized report (Insight report) generated by a third-party laboratory, Microba [27].

### 2.8. Changes to Protocol (Eligibility and Recruitment)

This study was initially targeted at pregnant women with a history of GDM (but free from diagnosis at enrolment) who were birthing at either one of two small metropolitan, public birthing facilities (study sites). However, after 3 months of recruitment, only one woman had enrolled to participate (the target was 12 at that timepoint). The study team then made changes to both the eligibility criteria and the recruitment method to improve recruitment rates (as outlined earlier). At the time of submitting this publication, recruitment has been open for eight months and 62 women have enrolled.

### 2.9. Intervention

The intervention is a multi-component dietary intervention that has been co-designed with mothers who have lived experience of GDM. It is expected that the co-design process will increase the intervention’s feasibility and acceptability and assist with the behavior change technique selection through a ‘behavior diagnosis’. The results of this process will be reported elsewhere. The objective of the intervention is to educate, motivate, and empower pregnant mothers and equip them with the skills and self-efficacy to make dietary changes that target the gut microbiota. The target behavior is *eating for gut health.* The women enrolled in the study receive one initial counseling session via telehealth and two follow- up support sessions (phone call or telehealth), as well as weekly text messages, supporting educational material and resources, and access to a private Instagram page and a private Facebook support group. To better describe each of the intervention components and in line with the Medical Research Council’s recommendation, the TIDieR Checklist [28] has been used and is available as Supplementary Material.

#### 2.9.1. Dietary Recommendations

The target behavior, ‘*eating for gut health*’ is described based on three main healthy gut diet principles:

1. Include a variety of plant foods (minimally processed) at each meal;
2. Reduce the intake of ultra-processed foods and foods high in saturated fat;
3. Eat naturally fermented foods.

Counseling is undertaken by an Accredited Practicing Dietitian, who is specifically trained to deliver the dietary intervention, in line with the Australian Dietary Guideline recommendations for pregnant women [29].

It is anticipated that women who consume a greater diversity of plant-based and fermented foods per week will have more diverse gut microbiota profiles compared with women who have low diversity and consume no fermented foods [23,30]. High intake of fiber and lower intake of ultra-processed foods and foods that are high in saturated fat is expected to result in lower levels of Gram-negative bacteria, such as the genus-level bacteria *Parabacteroides, Prevotella*, *Haemophilus* and *Desulfovibrio* [17], and higher levels of Short-Chain Fatty Acid-producing bacteria, including the species *R. Bromii* and *A. Putredinis* [31,32].

#### 2.9.2. Intervention Procedures

Following consent and enrolment, women are sent a link to book a telehealth appointment with the intervention dietitian. Ideally, the first appointment (approximately 45 to 60 min in length) is attended within 1 to 2 weeks of enrolment and must occur before 18 weeks gestation (but not before 12 weeks gestation). Intervention content and procedures are outlined in Supplementary Table S1: Item 2. Women are encouraged to set three dietary behavior goals at the initial appointment, in alignment with the three Healthy Gut Diet recommendations. At each follow-up, progress towards the goals is reviewed with the dietitian and revised where necessary. For intervention fidelity, each appointment will follow a pre-defined script with a fidelity checklist to ensure all participants receive the same information. The study lead, also an Accredited Practicing Dietitian, randomly selects two counseling sessions per month to attend as an observer to further assess fidelity of intervention delivery.

#### 2.9.3. Behavior Change Techniques

A range of behavior change techniques (BCTs) have been integrated into the intervention, largely informed by the intervention co-design process. These have been mapped to a published taxonomy [33] and are shown in Table 1.

### 2.10. Usual Care (Control Group)

Participants in both groups undertake all usual antenatal appointments according to their selected model of antenatal care. The control group also receives a 3 min video providing brief advice on general healthy eating in pregnancy according to the Australian Dietary Guideline [29] recommendations and a link to the electronic version of the Guidelines. Women in the control group complete all data collection tasks but do not see the study dietitian or receive additional support such as text messages or access to private social media pages.

### 2.11. Data Collection

Data are collected at various time points, as shown in Table 2. Following consent, enrolment and randomization, all women complete online questionnaires at the beginning of the study (within 1 week of enrolment) and at 36 weeks gestation. The women will also be invited to complete a follow-up questionnaire at 12 weeks postpartum. The questionnaires, delivered via REDCap, will collect information on:

1. Demographic data;
2. Dietary intake (using the Short Dietary Questionnaire);
3. International Physical Activity Survey [34];
4. Edinburgh Postnatal Depression Score [35];
5. Previous GDM diagnosis and management (where relevant);
6. Intake of dietary supplementation, probiotics and antibiotics;
7. Self-reported height and pre-pregnancy weight.

A separate paid questionnaire, known as the Australian Eating Survey (AES) (hosted by the University of Newcastle), is sent from the survey dashboard upon enrolment and at 36 weeks gestation [36]. The AES is a semi-quantitative Food Frequency Questionnaire validated for use in Australian Adults [36]. It is a self-administered tool used to collect information on estimated dietary intake over the last 3–6 months through 120 dietary items assessing frequency of consumption, from never to ≥seven times per day [36]. It provides information on food, food groups, macro and micronutrient consumption, and alignment with the Australian Dietary guidelines [36].

Dietary behavior goals set by women in the intervention group will be recorded at the initial counseling appointment by the dietitian. Women will be contacted at two time points in trimester 2 and 3 to complete a 24 h recall with a trained dietitian, using the multi-pass method. Maternal and infant outcome data, including GDM diagnosis and gestational weight gain, will be collected from medical records. At baseline and 36 weeks, women will provide stool samples using a self-collection sample kit provided by Microba Life Sciences [27]. Microba is a precision microbiota science company that will be providing high quality taxonomic, functional data and sample quality metrics from the microbiota sampling kits. Microba use shotgun metagenomic sequencing using the NovaSeqTM 6000 (Illumina, San Diego, CA, USA) with 2 × 150 bp paired-end chemistry at a target depth of 3 Gb per sample. Species classification will be determined with the Microba Community Profiler (v2), which uses a curated set of genome sequences from the public domain and Microba proprietary data (Microba Genome database, MGDB) a validated pipeline for producing comprehensive and accurate species profiles of human gastrointestinal samples [27]. All data (excluding microbiota data) will be collected and stored using the online data capture tool called REDCap 13.10.6.

### 2.12. Ethical Considerations and Monitoring

The study was approved by the Metro North Committee A Human Research Ethics Committee (HREC/2021/QRBW/82350) and the University of Queensland Human Ethics Committee. Current protocol version 1.8. 17 March 2023. As this study a dietary intervention only with no medication or supplementation, minimal harm is expected. No data monitoring or trial auditing procedures were developed. Any adverse events will be reported to the approving Human Research Ethics Committee in accordance with reporting requirements of the HREC and The University of Queensland.

### 2.13. Statistical Methods

The study results will be reported as per the Consolidated Standards of Reporting Trials (CONSORT) guidelines [37]. Results will be reported using an intention-to-treat approach, so all women in the intervention arm will be included in the primary analysis. In a sensitivity analysis, a per protocol approach will be used. Data from women who did not complete at least two of the three dietary counseling sessions will be excluded from this analysis. For the primary outcome (Section 2.13.1), comparisons between the groups using the binary outcome of GDM or no GDM diagnosis will be conducted using chi-square analysis. Logistic regression models will be used to adjust for potential confounders, decided a priori using causal diagrams. Confounders will include age, parity, pre-pregnancy BMI (categorical), weight gain above the Institute of Medicine recommendations based on pre-pregnancy BMI [38], assisted conception, and antibiotic use.

#### 2.13.1. Primary Outcome

The primary outcome will be the development of GDM according to the methods and diagnostic criteria outlined in the Queensland Clinical Guidelines [39], which are based on the International Association of Diabetes in Pregnancy Study Group recommendations [40].

#### 2.13.2. Secondary Outcomes

The following items will also be evaluated and reported (Table 2).

#### 2.13.3. Dietary Intake

Long-term maternal dietary intake, measured at baseline, 36 weeks, and 12 weeks, postpartum will be measured using the AES to report on intake of the core food groups, intake according to the Australian Recommended Food Score (ARFS) [41] and energy, fiber, and macronutrient intakes. Short-term (2-week) measures of dietary intake using a version of the Simplified Dietary Questionnaire (SDQ), first modified by Dawson et al. [25] to include prebiotic and probiotic foods will be included and cross-checked against 24 h diet recalls collected at four time points across trimesters 2 and 3. Maternal dietary quality and variety will be evaluated by applying the ARFS and healthful plant-based diet index [42], measured at baseline, trimesters 2 and 3, and 36 weeks gestation. The AES and 24 h dietary recalls will be used to report on the intake of core food groups (servings/day) and daily macro and micronutrients, including energy (kJ), protein (grams), total fat (grams), saturated fat (grams), and fiber (grams). The diet recall data will be entered into an Australian-based program known as FoodWorks [43], which provides the most comprehensive record of foods and food products available in Australia. FoodWorks provides data on energy (kJ), fiber and macro and micronutrient intakes. The modified SDQ will be scored to provide baseline, 36-week and 12-week postpartum data on diet quality and intake of probiotic and prebiotic foods. A Goldberg ratio of ≤0.9 will be used to identify potential under-reporters of energy intakes [44,45].

#### 2.13.4. Gut Microbiota

A between-group difference in microbial alpha diversity (Shannon Diversity Index), inverse Simpson index, Chao1, observed species and phylogenic diversity, relative abundance of operational taxonomic units measured at baseline and 36 weeks, as well as between-sample beta diversity, differences in taxa abundance, and functional capacity between groups and across pregnancy will be analyzed. Differential abundance analysis will be determined at a species level; this will be conducted between groups (intervention and control) and at an individual level. We will be evaluating both the group changes to the microbiota and individual changes from baseline. The influence of the *Healthy Gut Diet* on gut microbiota will be assessed by adjusting for ‘low’ or ‘high’ adherence to the dietary principles using the plant-based index [42] and ARFS [41] as both dichotomous and continuous variables. A sensitivity analysis will be undertaken to understand the impact of antibiotic use during pregnancy on changes to gut microbiota.

#### 2.13.5. Health Outcomes

Relevant pregnancy and infant outcomes include gestational age at delivery, mode of delivery, preterm birth, pregnancy-induced hypertension, polyhydramnios, edema, large-and-small-for-gestational age, neonatal hypoglycemia, and admission to a special care nursery.

Gestational weight gain recorded in medical records (and cross-referenced against self-reported weight at the 36-week survey) will be calculated by subtracting pre-pregnancy weight from the last recorded pregnancy weight. Gestational weight gain will also be classified as above, below, or within recommendations according to the Institute of Medicine cut-offs [38] based on pre-pregnancy body mass index.

### 2.14. Sample Size

The initial sample size calculation was based on the original eligibility criteria (previous GDM diagnosis), which assumed a background incidence of 50% (35 to 80% of women with a prior GDM diagnosis will develop it in subsequent pregnancies [46]). The original recruitment target of 40 women per group provided 80% power to detect a difference of 50% diagnosis rate in the control group and 25% in the intervention group (with a two-sided 5% significance level). However, as the changes to protocol outlined in Section 2.8 to include women with one or more risk factors for GDM have variably altered the background incidence of GDM, it is difficult to estimate the sample size needed for this trial. Therefore, a target of 120 women has been set on the basis that this is a pilot RCT, considering how many participants we can feasibly recruit within the study’s budget and timeline.

## 3. Results

Recruitment commenced in January 2023 and will continue until 120 women have been recruited or until April 2024 (whichever comes first). Data will be analyzed following completion of all data collection for the final participant, which is anticipated to occur by October 2024. At the time of manuscript submission, 503 women had registered their interest in the study and 74 women had consented and were randomized into either control (*n* = 36) or intervention (*n* = 38) groups.

## 4. Discussion

There is good evidence to suggest that the gut microbiota play a role in the development of GDM. To the best of our knowledge, there have been no dietary interventions aimed at improving ‘gut health’ in pregnancy for the purpose of preventing GDM. One prospective observational study involving 41 women with GDM showed that participants who were adherent to standard GDM nutritional recommendations had better metabolic and inflammatory outcomes and exhibited a significant decrease in the Bacteroides species [22]. To date, there have been no prospective trials used to evaluate the impact of dietary modulation of the gut microbiota and its role in the development of GDM. This pilot RCT will evaluate the effectiveness of the *Healthy Gut Diet* in preventing GDM in women with one or more risk factors and will examine the relationship between dietary intake and the gut microbiota during pregnancy.

Although this is the first RCT to target the gut microbiota in pregnant women with the aim of preventing GDM, there was a recent dietary intervention trial targeting the gut microbiota in pregnant women with the aim of improving maternal diet [25]. In this study, women in the intervention group significantly improved the quality and variety of their diet and their intake of pre- and pro-biotic foods from baseline to follow-up compared to the control group [25]. The impact of such dietary changes on maternal and infant gut microbiota composition for this study has yet to be determined.

Studies investigating changes in the gut microbiota during pregnancy are limited and have yielded mixed results regarding the remodeling of the microbiota during pregnancy. Some studies report dramatic shifts to the gut microbiota across pregnancy trimesters [47,48], whilst others suggest a relatively stable microbial profile [49,50,51]. Yang et al. linked variations in composition and function with GWG, age, residency status, pre-pregnancy BMI, and gestational diseases [51], suggesting that individual heterogeneity is the major force that shapes the microbiota in pregnancy, as opposed to gestation-age variations. By adequately controlling for confounding factors, we anticipate that gestational age variations will have little impact on the result.

Designing the intervention with the input of women who have lived experience of GDM to include a behavioral ‘diagnosis’ and carefully selected BCTs was important for improving applicability and adherence to the nutrition education and target behaviors. One concern associated with dietary interventions is ensuring the separation of any null results from participants who are not responding to the proposed dietary mechanisms versus not implementing the dietary recommendations due to poor dietary education/intervention design or barriers to adherence. We expect that the dietary intervention will be effective at increasing intake of dietary fiber and pre- and probiotic foods while reducing the intake of ultra-processed and foods high in unsaturated fat in the intervention group.

The strengths of the present study include its RCT study design, inclusion criteria targeting women with risk factors for developing GDM, strict exclusion criteria, robust co-designed intervention that includes pre-specified behavior change techniques, advanced (shotgun) microbial analysis techniques, and an a priori analysis plan. A major limitation is that the broadened eligibility criteria introduces the possibility that the study will be underpowered to demonstrate statistically significant changes between groups for the primary outcome (GDM diagnosis). Previously, the study was sufficiently powered to detect a 25% decrease in GDM diagnosis in the intervention group, as seen in other dietary GDM prevention trials for women with previous GDM. With the introduction of additional GDM risk factors, it is difficult to predict the expected reduction in GDM diagnoses, as the background incidence of GDM is variable and dependent on the risk factors present in participating women. This pilot trial will serve as a useful estimate for future studies and sample size power calculations. An additional concern is that women randomized into the control group will independently seek out information on improving gut health through diet. However, based on our robust dietary intake data collection methods, we expect we can both adjust for the presence of dietary confounders and/or undertake sub-group analyses to control for any bias introduced due to women in the control group independently altering their diets.

The findings of this study will advance the current knowledge in this field in terms of using a dietary approach to modulate the pregnant gut microbiota with the aim of preventing GDM, as well as behavior change theory. Potential outcomes (other than the development of GDM) may include fewer pregnancy complications, pregnancy weight gain within recommendations, and improved dietary intake. The postpartum follow-up for this study is designed to look at barriers and enablers of adherence to a Healthy Gut Diet, as well as longer-term sustainment of dietary interventions enhanced using BCTs.

## Figures and Tables

**Table 1:** Behavior change techniques (BCTs) [33] used in the Healthy Gut Diet Study.

BCT Category	BCT	Dietitian Education	SMS
Goals and planning	Goal setting (behavior) (1.1)	✔	✔
	Problem solving (1.2)	✔	✔
	Goal setting (outcome) (1.3)	✔	
	Action planning (1.4)	✔	✔
	Review behavior goal(s) (1.5)	✔	✔
	Discrepancy between current behavior and goal (1.6)	✔	
	Behavioral contract (1.8)	✔	
	Commitment (1.9)	✔	
Feedback and monitoring	Monitoring of behavior by others without feedback (2.1)	✔	
	Feedback on behavior (2.2)	✔	
	Self-monitoring of behavior (2.3)		✔
Social Support	Social support (unspecified) (3.1)	✔	✔
	Social support (practical) (3.2)	✔	
	Social support emotional (3.3)		✔
Shaping knowledge	Instruction on how to perform the behavior (4.1)	✔	✔
Natural consequences	Information about health consequences (5.1)	✔	✔
Association	Prompt/cues (7.1)		✔
Repetition and substitutions	Behavior substitution (8.2)	✔	✔
	Graded tasks (8.7)		✔
Comparison of outcomes	Credible source (9.1)	✔	
	Pros and Cons (9.2)	✔	
	Comparative imagining of future outcomes (9.3)	✔	
Reward and threat	Future punishment (10.11)	✔	
Regulation	Reduce negative emotions (11.2)		✔
Antecedents	Restructuring the physical environment (12.1)		✔
Identity	Identification of self as a role model (13.1)		✔
	Framing/reframing (13.2)	✔	✔

BCT—Behavior change technique.

**Table 2:** Data collection and time points for the Healthy Gut Diet Study from baseline to 36 weeks gestation.

Outcome Measure	Baseline (11 to 18 Weeks)	14 to 27 Weeks	28 to 40 Weeks	36 Weeks
GDM diagnosis (primary outcome)	✓ (Eligibility)		✓	✓
GDM management	✓		✓	✓
Anthropometric data	✓			✓
Demographic information	✓			
Medical history	✓			
Depression screening	✓			✓
Physical activity	✓			✓
Dietary intake	✓	✓	✓	✓
Microbiota analysis	✓			✓
Maternal and infant outcomes				✓
Satisfaction with care				✓

## Data Availability

The data presented in this study are available on request from the corresponding author. The data are not publicly available due to privacy restrictions and conditions of ethical approval.

## Supplementary Materials

The following supporting information can be downloaded at: https://www.mdpi.com/article/10.3390/nu15214653/s1, Table S1: The Healthy Gut Diet for preventing gestational diabetes mellitus Study described as per the Template for Intervention Descriptions and Replication (TIDieR) checklist; Table S2: Intervention content and procedures for the Healthy Gut Diet for preventing gestational diabetes mellitus Study. References [52,53,54,55,56,57,58,59,60,61,62] are cited in the supplementary materials.
