# Supplementary material for: Preventing Gestational Diabetes with a Healthy Gut Diet: Protocol for a Pilot, Feasibility Randomized Controlled Trial
Source: Nutrients. 2023 Nov 2;15(21):4653. doi: 10.3390/nu15214653 (PMC10649061; doi:10.3390/nu15214653)
Supplement: Supplementary file 1 [file nutrients-15-04653-s001.zip › nutrients-2639691-supplementary.pdf]

Supplementary Table S1: *The Healthy Gut Diet for preventing gestational diabetes mellitus Study* described as per the Template for Intervention Descriptions and Replication (TIDieR) checklist[1]

TIDier Item	Description
**1. Brief Name**<br>Provide the name of phrase that describes the intervention	The *Healthy Gut Diet* for preventing gestational diabetes mellitus (GDM) Study
**2. Why**<br>Describe the rationale, theory or goal of the elements essential to the intervention	The intervention consists of 6 elements:<br>• The Healthy Gut Diet: The gut microbiota plays a role in glycaemic control in GDM and other metabolic factors that can influence future progression to type 2 diabetes [2]. A *healthy gut* diet is abundant in fibre-rich, prebiotic whole foods found exclusively in vegetables, fruit, wholegrains, legumes/lentils, nuts and seeds[3]. The positive effects of a plant-focused diet are well reported [4, 5].<br>• Individualised dietary counselling: and tailoring dietary interventions for women in pregnancy can positively impact diet quality[6, 7], gestational weight gain[8, 9], and the development of GDM [6, 10].<br>• Telehealth delivery: Lifestyle interventions in pregnancy are often intensive and appointments are difficult to attend, particularly for mothers with one or more children. Telehealth/ video call interventions in pregnancy are effective in improving a range of obstetric outcomes [11] and can be used to deliver dietary interventions by an Accredited Practising Dietitian.[12]<br>• Behaviour change techniques: are important to enable dietary change [13, 14]. One approach is incorporating techniques from a published taxonomy[15], guided by the Behaviour Change Wheel[16].<br>• Resources, tools and other supporting educational material: particularly when co-designed with the target population can enhance dietary interventions.<br>• Embedded support both from program administration (text messages with embedded BCTs) and peer support (through a program administered Facebook group) to support behaviour change can enhance adherence.
**3. What: Materials**<br>Describe any physical or informational materials used in the intervention, including those provided to participants or used in the intervention delivery or in training of intervention providers. Provide information on where the materials can be accessed	Counselling sessions guided by script and PowerPoint presentations:<br>• Initial counselling session (45 – 60 minutes)<br>o Healthy Gut diet essentials education<br>o SMART goal setting<br>o Intervention (program) timeline<br>o FAQs<br>o Diet recall<br>• 2 week follow up (20 – 30 minutes)<br>o Revision of SMART goals<br>o Troubleshooting<br>o Intervention (program) timeline<br>o Diet recall<br>• 28 Week follow up (20 minutes)

	o SMART Goal revision<br>o Check GDM status<br>o Diet recall<br>Participant handbooks<br>• The Healthy Gut Diet Participant Handbook (initial)<br>o Goal setting and action planning templates<br>o Australian guide to healthy eating,<br>o Common barriers and solutions<br>o FAQs<br>o 4 week dietary plan<br>• Healthy Gut Plus+ Handbook (2 week follow up)<br>o Plus+ elements to building a healthy gut (prebiotic foods, exercise, sleep, stress)<br>o Reading food labels<br>o money saving tips healthy gut diet, overcoming challenges, prebiotic foods, exercise, sleep and stress<br>• Continuing After Pregnancy Handbook (36 weeks)<br>Participant recipe book<br>• 30 family and budget friendly recipes, designed in line with food safety recommendations in pregnancy and based on foods famililar to popualtion<br>• Recipes colour coded to match meal type (i.e breakfast is orange and lunch is blue)<br>Telehealth Session manuel<br>• Session check list for fidellity<br>• Initial session assessment template, collecting data on pariety, GDM history and current diet<br>• Session notes template<br>Participant plant points checklist<br>• Printable checklist of plant foods<br>Social media pages<br>• Instigram and facebook pages providing alternate format of eductaional materials, recipes and motivational messaged in additon to an anevue of social support.<br>Text messaging<br>• Weekly motivational/ informative messages (minimum 1 per week, up to 3 per week)<br>• Appointment and key activities reminders
4. **What: Procedures** Describe each of the procedures, activities and/or processes used in the intervention, including any enabling or support activities	Individual diet counselling<br>• 1 x initial diet counseling delivered via telehealth video call. The goal of thes sessions is to educate and increase adherance to healthy gut diet. Three key pillars are described: eat plenty of different plant foods at each meal, reduce ultra processed foods and foods high in saturated fats and eat naturally fermented foods.<br>• Assesment of participants understanding of a healthy gut and a healthy gut diet, corrections made where needed.<br>• Introduce and define SMART goals<br>• Set and agree on three SMART goals for the three key pillars

		- Following couseling session, participant emailed a summary of the three agreed SMART goals, participant resources and contact information. Follow up dietary couseling 2 x follow up dietary couseling sessions deliverd by phone call or telehealth. - Includes assesment of previously set SMART goals, identification of new goals, assesment of barriers and problem solving. - Optional discussion of Healthy gut plus+ materials Text message support 1-3 text messages delivered per week, providing motivational messages, links to resources, educational information, integrated with behaviour change techniques and co-designed with women with a lived experience of GDM. Group support - Optional private facebook and instagram groups for peer support, moderated by study lead.
**5.**	**Who Provided**<br>For each category of intervention provider (e.g., psychologist, nursing, assistant), describe their experience, background and any specific training given	Intervention provided by a single Accredited Practicing dietitian (APD) with one and a half years' experience working in maternity and GDM. They completed training in the behaviour change technique taxonomy V1 and specific training for intervention delivery.
**6.**	**How**<br>Describe the modes of delivery (e.g. face to face or by some other mechanism, such as the internet or telephone) of the intervention and whether it will be provided individually or in a group	Initial individual sessions are delivered by telehealth (video call); follow up sessions delivered via telehealth or phone call depending on participant preference; 1-3 text messages per week, participants can opt out; optional Facebook and Instagram private pages, usage dependent on participant engagement with the platform<br><br>For women unable to attend first telehealth session, three videos, designed to replicate first education session can be provided, followed by follow up phone call to set and agree on SMART goals.
**7.**	**Where**<br>Describe the type of location where the intervention occurred, including any necessary infrastructure or relevant features	The intervention is delivered using telehealth calls to the participants preferred location (home, work, other).<br>Participant requirements - Device with internet connection with webcam and microphone capabilities (phone, laptop, ipad or desktop). Dietitian requriments - Internet and VPN connetion, and device with webcam and microphone capabilities - Private room
**8.**	**When and how much**<br>Describe the number of times the intervention was delivered and over what period of time including the number of sessions,	The intervention is delivered over an 18–24-week period, from the first trimester of pregnancy (12-18 weeks) to 36 weeks.<br><br>There are three planned individual counselling sessions. The first one lasts ~1hour, followed by two 20-40 minutes follow up sessions. The first session is scheduled at baseline (12-18 weeks

	their schedule, duration, intensity or dose	gestation), followed by a review appointment 2 weeks later and a third at 28 weeks gestation.<br>Optional additional appointments for SMART goal review and questions during the intervention.
**9.**	**Tailoring**<br>If the intervention was planned to be personalised, titrated or adapted, then describe what, why, when and how	SMART goals and dietary recommendations personalised and tailored to participant's choice of goal, capability, and current diet.<br>Setting of new goals at intervention follow up dependent on participant choice and progress with previously set goals.
**10.**	**Modifications**<br>If the intervention was modified during the course of the study, describe the changes (what, why, when and how)	N/A
**11.**	**How Well: Planned**<br>If intervention adherence or fidelity was assessed, describe how and by whom, and if any strategies were used to maintain or improve fidelity, describe them	Training<br>• Telehealth resources- technology instrustions<br>• Standard session protocol and guide, guided by BCW taxonomy of behaviour change<br>A Redcap database developed to capture delivery data<br>• Recruitment data;<br>• Session attendance and duration;<br>• Adherence to session protocol;<br>• Fidelity checklist of components;<br>• Adverse events and questions asked<br>Random sampling of intervention delivery<br>• Study lead will randomly attend 2 sessions per month to assess fidelity<br>Process evaluation survey<br>• Modified quality of prenatal care questionnaire included in 36-week questionnaire<br>• Qualitative interviews for detailed feedback

References

1. Hoffmann TC, Glasziou PP, Boutron I, Milne R, Perera R, Moher D, Altman DG, Barbour V, Macdonald H, Johnston M, et al: **Better reporting of interventions: template for intervention description and replication (TIDieR) checklist and guide.** *BMJ : British Medical Journal* 2014, **348:**g1687.
2. Fugmann M, Breier M, Rottenkolber M, Banning F, Ferrari U, Sacco V, Grallert H, Parhofer KG, Seissler J, Clavel T, Lechner A: **The stool microbiota of insulin resistant women with recent gestational diabetes, a high risk group for type 2 diabetes.** *Sci Rep* 2015, **5:**13212.
3. McDonald D, Hyde E, Debelius JW, Morton JT, Gonzalez A, Ackermann G, Aksenov AA, Behsaz B, Brennan C, Chen Y, et al: **American Gut: an Open Platform for Citizen Science Microbiome Research.** *mSystems* 2018, **3:**e00031-00018.
4. Kim H, Caulfield LE, Rebholz CM: **Healthy Plant-Based Diets Are Associated with Lower Risk of All-Cause Mortality in US Adults.** *J Nutr* 2018, **148:**624-631.
5. Medawar E, Huhn S, Villringer A, Veronica Witte A: **The effects of plant-based diets on the body and the brain: a systematic review.** *Transl Psychiatry* 2019, **9:**226.

6. Opie RS, Neff M, Tierney AC: **A behavioural nutrition intervention for obese pregnant women: Effects on diet quality, weight gain and the incidence of gestational diabetes.** *Australian and New Zealand Journal of Obstetrics and Gynaecology* 2016, **56:**364-373.
7. Dawson SL, Mohebbi M, Craig JM, Dawson P, Clarke G, Tang MLK, Jacka FN: **Targeting the perinatal diet to modulate the gut microbiota increases dietary variety and prebiotic and probiotic food intakes: results from a randomised controlled trial.** *Public Health Nutrition* 2020**:**1-13.
8. Robertson N, Ladlow B: **Effect of individual dietetic intervention on gestational weight gain and associated complications in obese pregnant women.** *Australian and New Zealand Journal of Obstetrics and Gynaecology* 2018, **58:**274-277.
9. Lamminpää R, Vehviläinen-Julkunen K, Schwab U: **A systematic review of dietary interventions for gestational weight gain and gestational diabetes in overweight and obese pregnant women.** *European Journal of Nutrition* 2018, **57:**1721-1736.
10. Koivusalo SB, Rönö K, Klemetti MM, Roine RP, Lindström J, Erkkola M, Kaaja RJ, Pöyhönen-Alho M, Tiitinen A, Huvinen E, et al: **Gestational Diabetes Mellitus Can Be Prevented by Lifestyle Intervention: The Finnish Gestational Diabetes Prevention Study (RADIEL): A Randomized Controlled Trial.** *Diabetes Care* 2015, **39:**24-30.
11. Cantor AG, Jungbauer RM, Totten AM, Tilden EL, Holmes R, Ahmed A, Wagner J, Hermesch AC, McDonagh MS: **Telehealth Strategies for the Delivery of Maternal Health Care.** *Annals of Internal Medicine* 2022, **175:**1285-1297.
12. Ferrara A, Hedderson MM, Brown SD, Ehrlich SF, Tsai A-L, Feng J, Galarce M, Marcovina S, Catalano P, Quesenberry CP: **A telehealth lifestyle intervention to reduce excess gestational weight gain in pregnant women with overweight or obesity (GLOW): a randomised, parallel-group, controlled trial.** *The Lancet Diabetes & Endocrinology* 2020, **8:**490-500.
13. Phillips JK, Higgins ST: **Applying behavior change techniques to weight management during pregnancy: Impact on perinatal outcomes.** *Preventive medicine* 2017, **104:**133-136.
14. Beulen YH, Super S, de Vries JHM, Koelen MA, Feskens EJM, Wagemakers A: **Dietary Interventions for Healthy Pregnant Women: A Systematic Review of Tools to Promote a Healthy Antenatal Dietary Intake.** *Nutrients* 2020, **12:**1981.
15. Michie S, Richardson M, Johnston M, Abraham C, Francis J, Hardeman W: **The behavior change technique taxonomy (v1) of 93 hierarchically clustered techniques: building an international consensus for the reporting of behavior change interventions.** 2013, **46:**81-95.
16. Michie S, Atkins L, West R: *The Behaviour Change Wheel: A Guide to Designing Interventions.* London: Silverback Publishing; 2014.

Supplementary Table S2: Intervention content and procedures for the *Healthy Gut Diet for preventing gestational diabetes mellitus Study.*

**Intervention group.**<br>*Target behaviour: Eating for gut health*
**First telehealth appointment, 40-60minutes** (Gestation week 11-18)<br>*If patient does not join telehealth session with visual PowerPoint presentation, a voice-over PowerPoint will be emailed accompanied by a phone call.*<br>**Slide 1-8**<br>**Purpose:** Education, skill building, rapport, motivation to change<br>**Objectives**:<br>• Build rapport and communicate the healthy gut diet journey.<br>• Communicate foundation knowledge about the link between a healthy gut and GDM.<br>• Explain what a gut healthy diet comprises.<br>• Inspire participants to want to act to improve their diet.<br>BCTs: Instruction on how to perform a behaviour (4.1); Information about health consequences (5.1); Credible source (9.1); Future punishment (10.11); Pros and Cons (9.2); Goal setting (outcome) (1.3); Framing/ Reframing (13.2); Comparative imagining of future outcomes (9.3)<br><br>**Slide 9-12 & Slides 25, 31, 36, 37**<br>Purpose: Education, skill building, goal setting<br>Objectives:<br>• Introduce goal setting as a core component of intervention.<br>• Introduce SMART goals.<br>• Agree on and set three SMART goals, one for each pillar of the HGD.<br>• Discussion of the barriers to changing behaviour.<br>• Affirm dietary goals and commitment to change the behaviour.<br>BCTs: Goal setting (behaviour) (1.1); 1.4 Action Planning (1.4); Problem solving (1.2); Behavioural contract (1.8); Commitment (1.9)<br><br>**Slide 13- 35**<br>**Purpose:** Education, practical skill building<br>**Objectives**:<br>• Complete dietary recall to understand current dietary behaviour.<br>• Provide detailed education of the components of diet that support gut health.<br>• Discuss techniques and practical techniques to align diet with a gut friendly diet, personalised based on current dietary intake.<br>• Discuss possible food substitutes to support a gut friendly intake.<br>BCTs: Discrepancy between current behaviour and goal (1.6); Instruction on how to perform a behaviour (4.1); behaviour substitution (8.2);<br><br>Slides 40-42<br>• Inform about private Instagram and Facebook page for women part of the HGD to support behaviour change.<br>• Inform about weekly text messages.<br>• Provide information on the best way to get in contact, and encourage contact<br>BCTs: Social support (unspecified) (3.1); Social support (practical) (3.2)<br><br>Follow up email after initial telehealth appointment.<br>**Purpose:** Provision of resources<br>**Objectives**:

- Provide resources: participant handbook, participant recipe book, plant points checklist
- Summarise the three SMART goals discussed and agreed on
-

BCTs: Adding objects to the environment (12.5);

Text message support week 1-2

BCTs: Goal setting (behaviour) (1.1); Instruction on how to perform the behaviour (4.1); Information about health consequences (5.1); Prompt/cues (7.1); Social support emotional (3.3); Focus on past successes (15.3)

Support telephone/ telehealth calls with scripted questions, 20-30 minutes, (gestation weeks 18-20 and 28 weeks)

Purpose: Individualised counselling, skill building, goal review, **e**ducation

Objectives:

- Diet recall
- Set and review own dietary goals.
- Reaffirm or affirm dietary goals and commitment to change the behaviour.
- Monitor dietary intake via diet recall and provide informative feedback on performance.
- Utilize diet recall to guide personalisation of behavioural goals and advice.

BCTs: Review behaviour goal(s) (1.5); Discrepancy between current behaviour and goal(s) (1.6); 2.2 feedback on behaviour (2.2); Social support (unspecified); Goal setting (behaviour) (1.1); Commitment (1.9); feedback on behaviour (2.2); monitoring of behaviour by others without feedback (2.1)

Text message support week 3-12

Action planning (1.4); Behaviour substitution (8.2); Framing/reframing (13.2); Graded tasks (8.7); Identification of self as role model (13.1); Information about health consequences (5.1); Information on how to perform a behaviour (4.1); Instruction on how to perform a behaviour (4.1); Problem solving (1.2); Reduce negative emotions (11.2); Restructuring the physical environment (12.1); Review behavior goal(s) (1.5); Social support (practical) (3.2); Social support (unspecified) (3.1); Social support emotional (3.3); Self-monitoring of behaviour (2.3); Focus on past successes (15.3); Self-talk (15.4);
